# Electrochemical Glucose Sensor Based on Dual Redox Mediators

**DOI:** 10.3390/bios15010009

**Published:** 2024-12-27

**Authors:** Changyun Quan, Yue Zhang, Yuanyuan Liu, Liping Wen, Haixia Yang, Xueqin Huang, Minghui Yang, Binjie Xu

**Affiliations:** 1 Hunan Provincial Key Laboratory of Micro & Nano Materials Interface Science, College of Chemistry and Chemical Engineering, Central South University, Changsha 410083, China; quanchangyun@cofoe.com; 2 Cofoe Medical Technology Co., Ltd., No. 816 Zhenghua Road, Changsha 410021, China; zhangyue@cofoe.com (Y.Z.); wenliping@cofoe.com (L.W.); yanghaixia@cofoe.com (H.Y.); huangxueqin@cofoe.com (X.H.); 3 Hunan Institute For Drug Control, Changsha 410001, China; hnyjyliuyuanyuan@163.com; 4 National Engineering Research Center of Personalized Diagnostic and Therapeutic Technology, Central South University, Changsha 410083, China

**Keywords:** electrochemical glucose sensor, dual redox mediator, Hexaammineruthenium trichloride, 1,10-Phenanthroline-5,6-dione

## Abstract

Electrochemical glucose sensor holds significant promise for the monitoring of blood glucose levels in diabetic patients. In this study, we proposed a novel electrochemical glucose sensor based on 1,10-Phenanthroline-5,6-dione (PD)/Ru(III) as a dual redox mediator. The synergistic effect of PD and Ru(III) was utilized to efficiently facilitate the electron transfer between the enzyme-active center and the electrode. Then, a commercial disposable electrochemical glucose sensor was constructed based on screen-printing electrodes. Experimental results indicated the synergy between PD and Ru(III) provided a promising electron transfer environment for a glucose dehydrogenase (GDH)-catalyzed glucose reaction. The sensor exhibits a linear glucose response range from 0.01 to 38.6 mmol/L, with a limit of detection (LOD) as low as 7.0 µmol/L and a sensitivity of 38 µA·L/(mmol·cm^2^). The accuracy of the sensor was further validated in spiked recovery tests of human venous blood samples. The glucose recovery rate was between 99.5% and 107%, with a relative standard deviation (RSD) of less than 3.2%. These results demonstrate that our sensor has high potential for commercialization and practical application in glucose monitoring.

## 1. Introduction

Diabetes mellitus is a formidable metabolic disorder [1]. Despite the strides made in modern medical technologies, the prevalence of diabetes is still rising, with more and more people relying on medication to control blood glucose level [2]. So, there is an increasing trend for portable monitoring devices to measure blood glucose levels [3]. Numerous electrochemical glucose sensors have been developed and commercialized utilizing biological enzymes (glucose oxidase and glucose dehydrogenase) and redox mediators for monitoring blood glucose concentration in diabetic patients [4]. However, glucose oxidase-based sensors [5] are sensitive to oxygen partial pressure, limiting their use primarily to glucose measurements of fingertip blood. In contrast, glucose dehydrogenase-based sensors [6] offer versatility, accommodating glucose measurements across various blood samples, including fingertip, venous, and arterial, due to their insensitivity to oxygen partial pressure. In addition, the performance of commercial electrochemical glucose sensors is significantly influenced by the choice of redox mediator. An ideal redox mediator should exhibit the following traits [7]: (1) intimate contact with the active site of the biological enzyme to enhance electron transfer efficiency; (2) superior thermal stability; (3) a low working potential that minimizes interference from other blood components; and (4) immunity to oxygen interference. Most of the commercially available electrochemical sensors for glucose use a single redox medium, which is always an iron-based compound. However, iron-based compounds as redox mediators exhibit significant drawbacks, such as low thermal stability, increased background current with temperature, and poor immunity to interference, which results in the preparation of sensors with a short validity period and diminished sensitivity and accuracy. Consequently, the selection of redox mediator is pivotal for the successful development of commercial glucose sensors.

Transition metal ruthenium complexes have been investigated as redox mediators with extremely low redox working potentials, which can effectively reduce or even eliminate the interference of easily oxidized substances from blood in the glucose assay [8]. Ruthenium complexes are also more thermally stable than conventional media such as potassium ferricyanide [9]. Leveraging these properties, numerous studies have been carried out to develop electrochemical glucose sensors using ruthenium complexes as redox mediators, demonstrating enhanced electron transfer kinetics and current signal responses. Deng’s team has crafted an interference-free glucose biosensor, capitalizing on the ultra-low redox potential (−0.15 V) of ruthenium complexes in conjunction with glucose oxidase [10]. Meanwhile, Nakabayashi ’s group studied dipolar ruthenium-ammonia complexes, which contain pyridinium ions, in facilitating glucose oxidation, revealing a high catalytic rate constant with glucose oxidase [11]. Despite these advances, ruthenium-based redox mediators, predominantly paired with glucose oxidase in sensor construction, remain susceptible to oxygen partial pressure, thus compromising sensor accuracy. Therefore, the development of an electrochemical glucose sensor that combines the oxygen-insensitive flavin adenine dinucleotide-dependent glucose dehydrogenase (FAD-GDH) with the exceptionally low potential of hexaammonium hexachloride ruthenium complex is of paramount importance. However, the research results by Loew indicated that a negligible response current was obtained during the oxidation of glucose, catalyzed by the fungal FAD-GDH and mediated by strongly positively charged Ru(III), suggesting that positively charged Ru(III) is not able to freely pass through the enzyme framework to reach the hidden active center [12]. Therefore, a neutral small molecule, serving as an intermediate mediator, is necessary to initially and efficiently convey electrons generated by the deeply embedded active center to Ru(III), and then to the electrode via Ru(III) redox reactions, thereby eliciting a current response.

Quinones serve as redox mediators for electrocatalysis or enzyme biosensors, due to their excellent chemical stability, favorable equilibrium potentials, electrochemical reversibility, and high reactivity to redox-active enzymes [13]. For example, 1,10-Phenanthroline-5,6-dione (PD), as a multi-purpose ligand, has two diimine nitrogen atoms suitable for metal ion binding, while the o-quinone portion can also be involved in redox reactions for electron storage and transportation [14]. Zor’s studies have demonstrated that PD, as a small molecule compound with no positive or negative charges, has a large electron self-exchange rate constant [15]. In addition, PD has high electron-acceptance efficacy due to the conjugated diketone in its structure, which allows it to undergo a direct redox reaction with the active center of enzymes.

**Figure 1:**
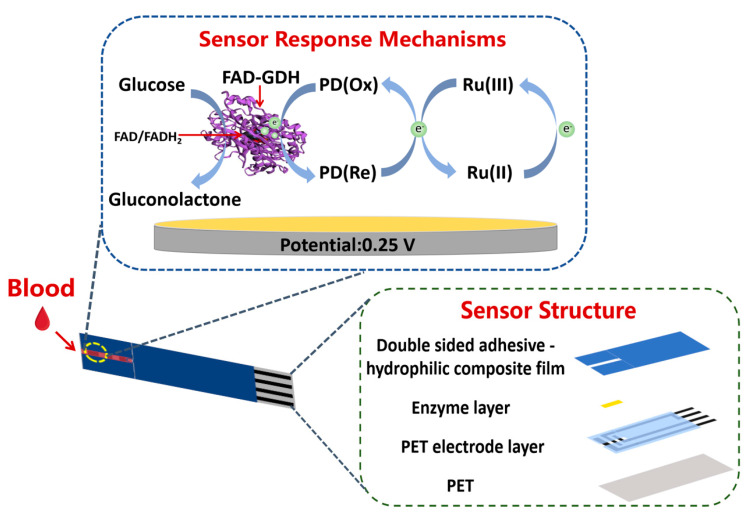
Schematic diagram of the structure and reaction mechanism of the electrochemical glucose sensor.

In this research, we developed a commercially viable, disposable electrochemical glucose sensor, based on FAD-GDH and screen-printing electrodes. This sensor capitalizes on the synergistic effects of PD and Ru(III) as redox mediators. As depicted in Figure 1, PD functions as the primary redox mediator, penetrating the protein structure to ferry electrons from the active center of the enzymes. Subsequently, Ru(III) serves as the secondary redox mediator, transferring the electrons from PD to the electrode via a redox reaction. The sensor is referred to as a PD/Ru(III) dual redox-mediated electrochemical sensor for glucose (PD/Ru(III) sensor), and its electrochemical performance for the detection of glucose in real samples is investigated. The development of the PD/Ru(III) sensor paves the way for innovative strategies in the commercialization of glucose sensors.

## 2. Experiment Section

### 2.1. Materials and Reagents

PD (97%) was purchased from Macklin. Glucose dehydrogenase (FAD-GDH, 596 U/mg), acetaminophen (PCM, 98%), uric acid (UA, 99%), ascorbic acid (AA, 99%), and glutathione (GHS, 98%) were obtained from Sigma Aldrich Trading (Shanghai, China) Co., Ltd. Glucose (C_6_H_12_O_6_, 98%), maltose (MAL, 98%), galactose (GAL, 99%), sodium chloride (NaCl, SR99.5%), disodium hydrogen phosphate (Na_2_HPO_4_, 99%), and sodium dihydrogen phosphate (NaH_2_PO_4_, 99%) were bought from Sinopharm Chemical Reagent (Shanghai, China) Co., Ltd. 3-morpholino propanesulfonic acid (MOPS, 99.5%), bis 3-morpholine propanesulfonic acid (MOPS, 99.5%), bis(2-hydroxyethyl) amino-tris(hydroxymethyl) methane (Bis-Tris, 99%), 3-[(hydroxymethyl) methylammonio] ethanesulfonic acid (TES, 99.5%), morpholine ethanesulfonic acid (MES, 99.5%), toluene sulfonylurea (TB, 98%), ibuprofen (IBU, 98%), dopamine hydrochloride (DH, 98%), heparin sodium (HS,180(units/mg)), cholesterol (CHO, 95%), creatinine (CR, 99%), xylose (XYL, 98%), iacodextrin (IDN, 98%), mannitol (MT, 99%), and sorbitol (SB, 99%) were purchased from Aladdin Biochemical Technology (Shanghai, China) Co., Ltd. Hexaammonium hexachloride ruthenium (Ru(III), 98%) was purchased from Energy Chemical (Shanghai, China) Co., Ltd.

### 2.2. Apparatus

The electrochemical performance of the PD/Ru(III) sensor was evaluated by an electrochemical workstation (CHI660E, Shanghai Chenhua Instrument (Shanghai, China) Co., Ltd.). The metabolites were analyzed by the blood-gas-oxygen-electrolyte-metabolite analyzer (ABL90, Radiometer Medical Equipment (Shanghai, China) Co., Ltd.). The partial pressure of oxygen in the blood was adjusted, and the glucose concentration in the samples was detected by a biochemistry analyzer (BS-360S, Mindray Bio-Medical Electronics (Shenzhen, China) Co., Ltd.), and the results were used as a reference standard.

### 2.3. Sensor Construction

Polyethylene terephthalate (PET) was selected as the substrate, and the carbon paste was printed on the substrate through a screen-printing technique to form electrodes. Then, a layer of insulating oil was printed to fix the working area, and finally a PET electrode layer was formed. For enzyme modification, 5 μL of enzyme solution was evenly drop-coated on the working area of the PET electrode layer, in which the enzyme solution consisted of 0.2 mg/mL PD, 30 mg/mL Ru(III), 2000 U/mL FAD-GDH, MES buffer solution (pH = 7.0), and 4 mg/mL hydroxyethyl cellulose. The enzyme layer was formed after oven drying (50 °C, 30 min), and then a double-sided adhesive-hydrophilic combination membrane was applied for siphoning of the sample. Finally, the portable disposable PD/Ru(III) sensor was prepared by cutting and drumming (with desiccant) (Figure 1). The sensor includes a working electrode and a counter electrode. The effective surface area of the working electrode is 1.2 mm × 1.3 mm = 1.56 mm^2^.

### 2.4. Evaluation of Electrochemical Performance of the Sensor

The PD/Ru(III) sensor was tested at different concentrations of glucose solution (1.5 mmol/L, 3 mmol/L, 6 mmol/L, 9 mmol/L, 12 mmol/L, 18 mmol/L). Chronoamperometry (CA) and current-time curve (I-t) methods were used to calculate the electrochemical parameters of PD/Ru(III)-mediated, FAD-GDH-catalyzed glucose processes. In addition, the PD/Ru(III) sensor was used to perform spiked recovery experiments on real samples to verify the accuracy of the sensor. The current values used in the experiments were taken from the 5ths of the I-t graph.

### 2.5. Blood Measurement

To prepare blood with different glucose concentrations, different amounts of glucose were mixed with the same volume of blood serum (ensuring that the blood erythrocyte pressure volume remained constant), allowing blood samples with different glucose concentrations to be obtained. Subsequently, each blood sample was divided equally into two portions, one portion was used to determine the actual concentration of blood glucose by a precision biochemical analyzer, and the other portion was used to detect the glucose concentration in the blood using a PD/Ru(III) sensor, and the working curve was used to perform a preliminary calibration.

## 3. Results and Discussion

### 3.1. Electrochemical Study of the Sensor

**Figure 2:**
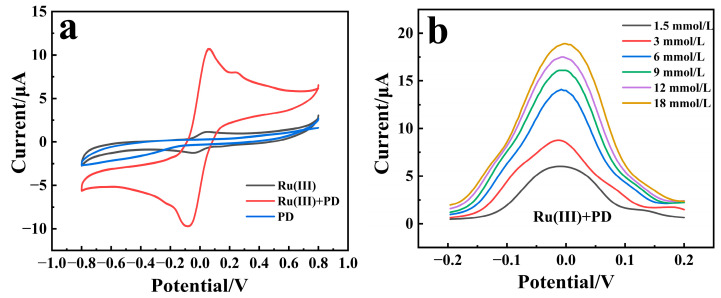

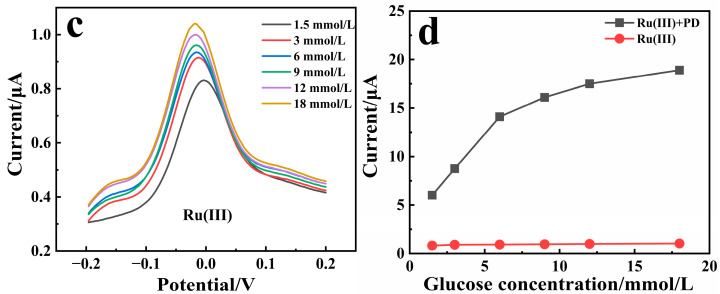
(**a**) CV plots of three sensors that were prepared using PD, Ru(III), or PD/Ru(III) as redox mediators and subjected to cyclic voltammetry scanning in 6 mmol/L glucose solution at a scan rate of 0.1 V/s. (**b**) DPV plot of PD/Ru(III) sensor and (**c**) Ru(III) sensor toward different concentrations of glucose. (**d**) Response currents of the Ru(III) sensor and the PD/Ru(III) sensor versus different glucose concentrations.

Three sensors were prepared using PD, Ru(III), and PD/Ru(III) as redox mediators, respectively, and were applied to detect glucose, utilizing cyclic voltammetry (CV) and differential pulse voltammetry (DPV). As shown in Figure 2a, cyclic voltammetry scans were performed on the three sensors for testing the 6 mmol/L glucose solution. It was observed that the CV curves of the sensors using only PD as the redox mediator do not have obvious redox peaks, which may be due to the low amount of PD utilized. This suggests that the mediator PD cannot transfer the electrons from the enzyme-catalyzed process to the electrodes directly. When Ru(III) and PD/Ru(III) were used as redox mediators, respectively, the CV curves of the corresponding sensors showed obvious redox peaks. The response current signal of the PD/Ru(III) sensor was much larger than that of the Ru(III) sensor, indicating that in the enzyme catalyzed glucose process, the mediator Ru(III) could only accept very few electrons in the enzyme catalytic process and transfer them to the electrode to trigger a small current response. In contrast, in the presence of both mediator PD and Ru(III), the PD can act as a bridge to redox reactions with the enzyme-active center within protein. After the double-bond of the conjugated quinone in its structure is opened, all the electrons are accepted and stored during the enzyme-catalyzed process [16], and then the bipyridine structure further transfers the stored electrons to Ru(III) through a complexation reaction [17]. Finally, the electrons are transferred to the electrode through the redox reaction of Ru(III) to trigger the response current. Therefore, the redox reaction process of glucose catalyzed by the PD/Ru(III) sensor can be deduced as the following Equations (1)–(4), in which the synergistic effect of PD and Ru(III) facilitates the electron transfer in the enzyme-catalyzed process and improves the response current signal of the sensor:

(1) $$\mathrm{Glucose}+\mathrm{FAD}-\mathrm{GDH}\to \mathrm{GluconicAcid}+{\mathrm{FADH}}_{2}-\mathrm{GDH}$$

(2) $${\mathrm{FADH}}_{2}-\mathrm{GDH}+\mathrm{PD}\to \mathrm{FAD}-\mathrm{GDH}+{\mathrm{PDH}}_{2}$$

(3) $${\mathrm{PDH}}_{2}+{2\mathrm{Ru}}^{3+}\to \mathrm{PD}+{2\mathrm{Ru}}^{2+}+{2H}^{+}$$

(4) $${\mathrm{Ru}}^{2+}\to {\mathrm{Ru}}^{3+}+e^{-}$$

Current responses of the PD/Ru(III) sensor and the Ru(III) sensor toward different glucose concentrations were measured by DPV and were shown in Figure 2b,c. As can be seen from Figure 2d, the current gradient variation of the PD/Ru(III) sensor for measuring different glucose concentrations is much higher than that of the Ru(III) sensor, further demonstrating that the synergistic effect between PD and Ru(III) significantly enhanced the sensitivity of the sensor.

### 3.2. Catalytic Activity Study of the Sensors

**Figure 3:**
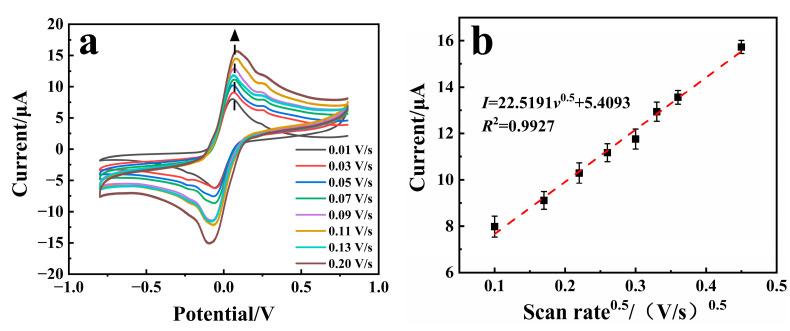

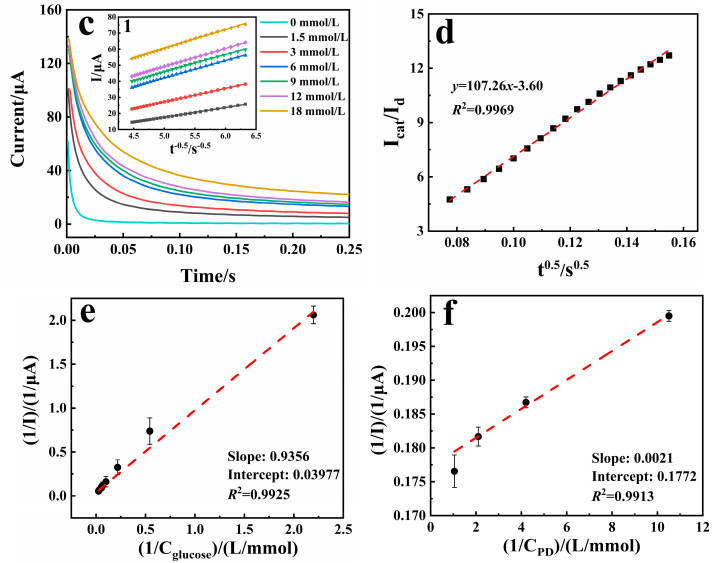
(**a**) Cyclic voltammograms of the PD/Ru(III) sensor at different scan rates. (**b**) The linear plot of the oxidation peak current versus the arithmetic square root of the scan rate. (**c**) Chronoamperometric current curves of the PD/Ru(III) sensor toward different concentrations of glucose; inset (**c1**) shows the linear relationship between the limiting current of the sensor at different concentrations of glucose and the inverse of the arithmetic square root of the electrolysis time. (**d**) The linear relationship between the ratio of the catalytic current to the basal current of the sensor and the arithmetic square root of the electrolysis time. (**e**) Double inverse plot of glucose concentration versus current. (**f**) Double inverse plot of medium concentration versus current.

Figure 3a shows the CV curves of the PD/Ru(III) sensor for the detection of 6 mmol/L glucose at different scanning rates. It can be seen that the current responses were obviously enhanced with a faster scanning rate, and the peak currents have a clear linear relationship with the arithmetic square root of the scanning rate (Figure 3b). These results demonstrated that the electrochemical catalytic oxidation of glucose on the PD/Ru(III) sensor is a diffusion-controlled process, so the diffusion coefficient (*D*_0_) and catalytic rate constant (*K*_cat_) of glucose can be calculated by using the single-potential chronoamperometry method [18].

Figure 3c shows the chronoamperometry profile of the sensor for detecting different concentrations of glucose. The current response under the diffusion control can be described by the Cottrell equation (Equation (5)), where *n* is the number of transferred electrons, *F* is the Faraday constant, *A* is the effective surface area of the working electrode (1.2 mm × 1.3 mm = 1.56 mm^2^), *t* is the electrolysis time, and *C*_0_ is the concentration of glucose. Inset Figure 3c(1) shows the linear correlation curves of the limiting current (*I*) versus the inverse of the arithmetic square root of the electrolysis time (*t*^−0.5^) for different concentrations of glucose, and an average *D*_0_ of 6.88 × 10^−6^ cm^2^/s was obtained through slope calculation.

(5) $$I=nFAC_{0}D_{0}^{0.5}\pi^{-0.5}t^{-0.5}$$

Similarly, the *K*_cat_ value of glucose can be determined by the chronoamperometry method and calculated by the equation (Equation (6)), where *I*_cat_ is the catalytic current in the presence of glucose and *I*_d_ is the basal current in the absence of glucose. The curve of *I*_cat_/*I*_d_ versus *t*^0.5^ is shown in Figure 3d, and the *K*_cat_ value of the sensor at 3 mmol/L glucose concentration can be calculated to be 1.22 × 10^9^ cm^3^/(mol·s).

(6) $$I_{\mathrm{cat}}/I_{d}=\pi^{0.5}(K_{\mathrm{cat}}C_{0}t{)}^{0.5}$$

Upon mechanistic investigation, as described above, the PD/Ru(III)-mediated FAD-GDH-catalyzed glucose-sensing process proximally conforms to the ping-pong enzymatic reaction mechanism [19], and the process can be quantified by the Michaelis–Menten equation. The Michaelis–Menten constant *K*_m_ reflects the affinity of the enzyme for the substrate, which can be evaluated by the linear relationship between the inverse of the current and the inverse of the substrate concentration (Figure 3e,f), according to the Lineweaver–Burk equation (Equation (7)). In the equation, *I* is the current, *I*_max_ is the maximum value of the current measured under the condition of saturated substrate, and *C* is the substrate concentration. The *K*_m_ value of FAD-GDH for glucose was calculated to be 14.3 mmol/L, and the *K*_m_ value of FAD-GDH for PD/Ru(III) was calculated to be 0.01 mmol/L.

(7) $$\frac{1}{I}=\frac{K_{m}}{I_{\max}}\times \frac{1}{C}+\frac{1}{I_{\max}}$$

The electrochemical parameters of the enzyme-catalyzed glucose reaction by different redox mediators are listed in Table 1. It can be found that the PD/Ru(III) sensor has a larger *D*_0_ value and a smaller *K*_m_ value compared with the sensors constructed by other redox media. These data proved that FAD-GDH has a better affinity with PD/Ru(III), and affirms that PD/Ru(III) provides a good mediating environment for the glucose catalyzed by FAD-GDH. It is also reaffirmed that PD has almost no spatial site-barrier and electrostatic repulsive interactions in the protein channel, which makes it easier for the uncharged small molecule mediator to approach the active center of FAD-GDH and further transfer electrons to the carbon electrode via Ru(III), to trigger a significant current response.

### 3.3. Optimization of Experimental Conditions

**Figure 4:**
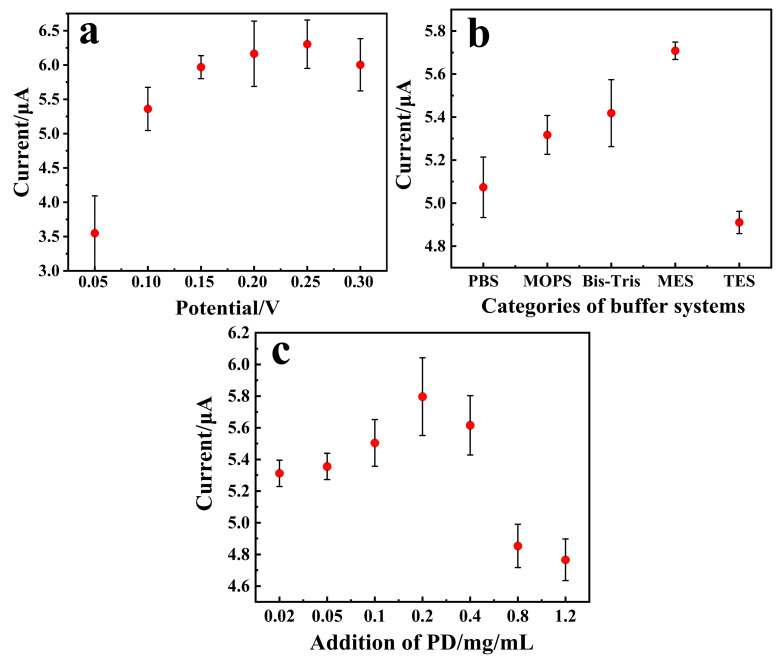
Effects of different parameters on the current signal of the PD/Ru(III) sensor. (**a**) Optimization of working potentials. (**b**) Optimization of different buffer systems, where the concentration of buffer systems was all 0.05 mol/L. (**c**) Optimization of PD content. The concentration of glucose used in the test was 6 mmol/L.

The working potential of the sensor, the buffer composition of the enzyme solution, and the loading amount of the redox mediator affects the sensitivity of the sensor, so the three parameters were optimized. As shown in Figure 2a, the oxidation peak of the PD/Ru(III) sensor is located between the potential range of 0.05 and 0.3 V. So, the operating potential of the PD/Ru(III) sensor was further optimized in this range (Figure 4a), and the response current value of the sensor reached maximum value when the operating potential was 0.25 V, which indicates that 0.25 V is the most suitable potential. The enzyme catalytic activity of FAD-GDH was maximum at pH 7.0 [24]. However, different types of enzyme buffer systems affect the response current of the sensor. Therefore, the effects of five different buffer systems on the response current of the sensor were investigated with the pH of the buffer system fixed at 7.0 (Figure 4b), in which the phosphate buffered saline (PBS) buffer solution was co-formulated with Na_2_HPO_4_ and NaH_2_PO_4_. The results showed that when MES was selected as the buffer system of the enzyme solution, the sensor has the highest sensitivity. This indicates MES was more conducive to the enzymatic reaction and electron transfer of the sensor. Therefore, MES was selected as the buffer system of the enzyme solution. Concentrations of redox mediators in the enzyme solution also influences the response current of the sensor. The sensor includes two kinds of redox mediators, Ru(III) and PD, of which Ru(III) is more expensive, so the concentration of Ru(III) was fixed to be 30 mg/mL, considering the cost control of the sensor. PD acts as a bridge for electron transfer during enzymatic reactions, its content directly affecting the current response of the sensor, so the effect of PD with a concentration of 0.02~1.2 mg/mL on the current signal of the sensor was investigated (Figure 4c). The results showed that the response current value of the sensor reached the maximum value when the PD content was 0.2 mg/mL. In summary, the optimal conditions for constructing the PD/Ru(III) sensor were as follows: the working potential of the sensor was 0.25 V, MES was chosen as the buffer system for the enzyme solution, and the PD concentration of the enzyme solution was 0.2 mg/mL.

### 3.4. Analytical Performance of the Sensor

**Figure 5:**
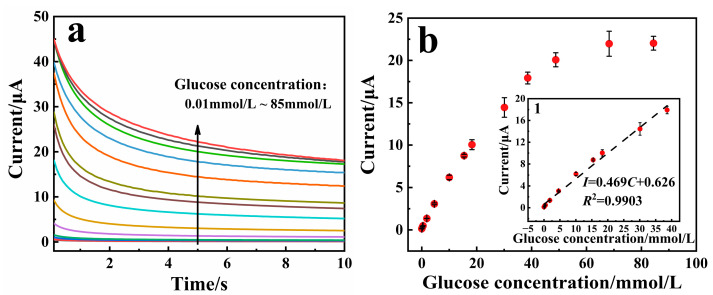
(**a**) I-t curve diagram of the PD/Ru(III) sensor for the detection of different glucose concentrations, where the glucose concentrations were 0.01, 0.06, 0.2, 0.5, 1.8, 4.6, 10.0, 15.4, 18.3, 30.1, 38.6, 48.8, 68.3, and 85 mmol/L. (**b**) Scatter plots of glucose concentrations corresponding to current values; inset (**b1**) shows the linear relationship between different glucose concentrations and currents. The above test samples were configured from human venous blood samples and calibrated for glucose concentration using a biochemistry instrument.

The responses of the PD/Ru(III) sensor toward glucose can be measured by the I-t method. Glucose test samples were configured from human venous blood samples and calibrated for glucose concentration using a biochemistry instrument. As shown in Figure 5a, the current values increased proportionally with the increase of glucose concentration. When the glucose concentration was in the range of 0.01~38.6 mmol/L, the current value at 5 s of the I-t curve showed a good linear correlation with the glucose concentration, with linear equation *I* = 0.469*C* + 0.626 (*R*^2^ = 0.9903, Figure 5b). The linear range of existing commercialized glucose sensors for detecting glucose concentration in blood is generally 1.1~33.3 mmol/L. Therefore, the PD/Ru(III) sensor constructed in this paper fully meets the requirements for commercialized practical applications. The limit of detection of the PD/Ru(III) sensor was calculated from the assay data to be 7.0 µmol/L (S/N = 3), and the sensitivity was 38 µA·L/(mmol·cm^2^). Table 2 lists the analytical parameters of the PD/Ru(III) sensor and other glucose biosensors. The PD/Ru(III) sensor was found to have a wider linear range and lower detection limit, therefore possessing a good prospect for commercialization and application.

**Figure 6:**
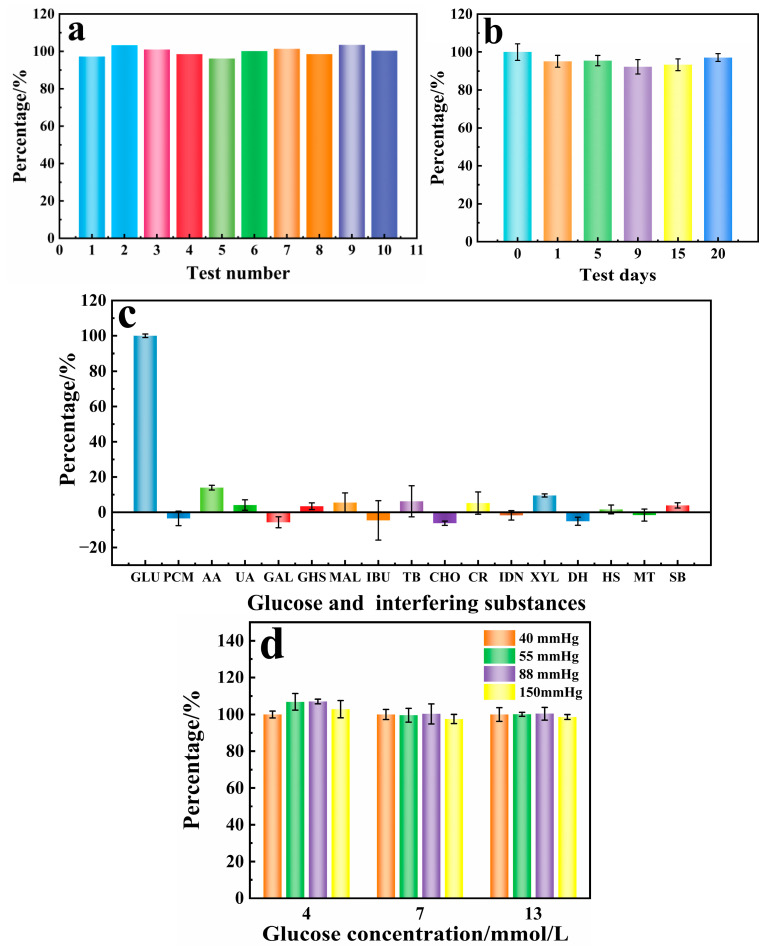
Reproducibility (**a**) stability (**b**) and selectivity (**c**) of the glucose sensor. (**d**) Effect of oxygen partial pressure toward glucose detection.

Good reproducibility and stability are important for commercialization of biosensors [33], so these characteristics of the PD/Ru(III) sensors were evaluated. The identical batch of 10 disposable PD/Ru(III) sensors were prepared, each of which was subjected to a single measurement of 6 mmol/L glucose blood sample (Figure 6a). The relative standard deviation (RSD) of the response current value was calculated to be 2.42%, which indicated that the PD/Ru(III) sensor had good reproducibility. The same batch of PD/Ru(III) sensors was prepared, and five measurements of 6 mmol/L glucose solution were performed on different days to assess the stability of the sensor. The results are shown in Figure 6b, where the PD/Ru(III) sensor still retained 97% of the initial response current value on day 20, indicating that the sensor has good long-term stability.

In actual blood samples, in addition to glucose, there are still a variety of interfering substances, which may generate microcurrents that affect the accuracy of the detection of glucose. Therefore, the selectivity of the sensor was studied. In this paper, the following 16 typical substances were selected for anti-interference experiments: ① Exogenous interfering substances (commonly used drugs, anticoagulants, and food-derived substances): PCM (20 mg/dL), AA (6 mg/dL), TB (100 mg/dL), IBU (50 mg/dL), DH (0.09 mg/dL), and HS (3000 U/L). ② Endogenous interfering substances (substances and metabolites commonly found in the body): UA (6 mg/dL), GHS (92 mg/dL), CHO (25 mg/dL), and CR (5 mg/dL). ③ Other interfering substances: MAL (200 mg/dL), XYL (200 mg/dL), GAL (15 mg/dL), IDN (1094.4 mg/dL), MT (500 mg/dL), and SB (500 mg/dL). As shown in Figure 6c, glucose can trigger a significant current signal, while other interfering substances only generate a small current response, indicating that the PD/Ru(III) sensor has a strong anti-interference capability. In addition, the oxygen partial pressure of the sensor was examined (Figure 6d), and the results showed that different oxygen partial pressures had very little effect on the results of the sensor for measuring the same glucose concentration, indicating that the constructed PD/Ru(III) sensor is not affected by the oxygen partial pressure, and thus the sensor can be applied to glucose detection in human fingertip blood, venous blood, or arterial blood.

### 3.5. Spiking Recovery Experiments in Real Samples (Venous Blood)

To validate the clinical application potential of this sensor, we performed spiking recovery experiments of glucose in actual human venous blood samples. Different concentrations of glucose were spiked into venous blood samples for testing. As shown in Table 3, the recoveries ranged from 99.5% to 107%, with a relative standard deviation (RSD) of <5% (n = 5), indicating that the sensor has good potential for clinical application.

## 4. Conclusions

In this paper, an electrochemical glucose sensor, based on a PD/Ru(III) dual redox mediator, was designed for glucose detection in blood samples. The synergistic effect of PD with Ru(III) was used to efficiently promote the electron transfer between the active center of FAD-GDH enzymes and the electrode, which significantly improved the sensitivity of the sensor. Experimental test results showed that the sensor has a wide linear range (0.01~38.6 mmol/L), good reproducibility, long-term stability, and anti-interference capability. The glucose spiked recovery of venous blood samples showed good measurement accuracy with recoveries ranging from 99.5% to 107%, and an RSD less than 3.2%. These properties provide stable prerequisites for the commercial application of this sensor.

## Figures and Tables

**Table 1:** Electrochemical parameters of different redox mediators for enzyme-catalyzed glucose reaction.

Redox Mediator	*D*_0_/cm^2^/s	*K*_m_/mmol/L	Reference
MWCNTs/PyBA-NMP	/	18	[20]
TTF/CNTs	/	21.4	[21]
Ferrocene monosulphonate	4.0 × 10^−6^	88	[22]
Fe(CN)_6_^3−^	6.7 × 10^−6^	65	[23]
PD/Ru(III)	6.88 × 10^−6^	0.01	This work

Notes: multi-walled carbon nanotubes (MWCNTs); 4-(1H-Pyrrol-1yl)benzoic acid (PyBA); n-methylphenazonium methyl sulfate (NMP); tetrathiafulvalene (TTF).

**Table 2:** Comparison of performance parameters of PD/Ru(III) sensor with other glucose sensors.

Sensor	Linear Range/mmol/L	LOD/mmol/L	Sensitivity/μA·L/(mmol·cm^2^)	Reference
MIP-PANI Paper Sensors	2.2~11.1	1.1713	/	[25]
SCT/PRG/CuNPs	0.1~0.6	0.025	1101.3	[26]
PGE glucose sensor	0.02~1.11	0.0027	22.05	[27]
AuNP-PPE	0.05~35	10.0	/	[28]
MWCNT/PyBA-GOx-NMP	10~12	0.3	12.1	[20]
PPD/(AuNP)PPCA-GOx	0.2~150.0	0.08	/	[29]
GR/PtNS/PD/GOx	16.5	0.198	10.1	[30]
GR/PtNS/PD/GOx/Ppy	39.0	0.561	5.31	
FIC/αPLL/GOx-SPCE	2.8~27.5	2.3	212.1	[31]
RuNCs/ITO	0.02~1.2	0.02	/	[32]
PD/Ru(III)	0.01~38.6	0.007	38	This work

Notes: molecularly imprinted polyaniline (MIP); polyaniline (PANI); scotch tape (SCT); physically rubbed graphene (PRG); nanoparticles (NPs); polyethylene terephthalate gold electrode (PGE); prepare printed paper electrodes (PPE); multi-walled carbon nanotubes (MWCNTs); 4-(1H-Pyrrol-1yl)benzoic acid (PyBA); pyrrole-2-carboxylic acid (PCA); graphite rod (GR); platinum nanostructures (PtNS); glucose oxidase (GOx); polypyrrole (Ppy); ferricyanide (FIC); α-poly-l-lysine (αPLL); screen-printed carbon electrodes (SPCE); ruthenium nanoclusters (RuNCs); indium tin oxide-coated on glass (ITO).

**Table 3:** Results of glucose detection in human venous blood samples using the PD/Ru(III) sensor (n = 5).

Real Samples	Initial Glucose/mmol/L	Spiked/mmol/L	Detected/mmol/L	Recovery/%	RSD/%
Human venous blood	5	2	7.0 ± 0.1	99.5 ± 2.1	2.2%
		5	10.3 ± 0.1	107 ± 2.4	2.2%
		10	15.6 ± 0.3	106 ± 3.5	3.2%
		15	19.5 ± 0.2	96.9 ± 1.3	1.3%
		20	25.3 ± 0.6	102 ± 2.9	3.0%

## Data Availability

The data presented in this study are all included in the manuscript.
